# The Difference between Rhizosphere and Endophytic Bacteria on the Safe Cultivation of Lettuce in Cr-Contaminated Farmland

**DOI:** 10.3390/toxics11040371

**Published:** 2023-04-13

**Authors:** Zheyu Wen, Qizhen Liu, Chao Yu, Lukuan Huang, Yaru Liu, Shun’an Xu, Zhesi Li, Chanjuan Liu, Ying Feng

**Affiliations:** 1 MOE Key Laboratory of Environment Remediation and Ecological Health, College of Environmental and Resource Sciences, Zhejiang University, Hangzhou 310058, China; 2 Livestock Industrial Development Center of Shengzhou, Shaoxing 312400, China

**Keywords:** chromium, microbial remediation, *Lactuca sativa* L., Cr-tolerant bacteria, Cr passivation

## Abstract

Chromium (Cr) is a major pollutant affecting the environment and human health and microbial remediation is considered to be the most promising technology for the restoration of the heavily metal-polluted soil. However, the difference between rhizosphere and endophytic bacteria on the potential of crop safety production in Cr-contaminated farmland is not clearly elucidated. Therefore, eight Cr-tolerant endophytic strains of three species: *Serratia* (SR-1~2), *Lysinebacillus* (LB-1~5) and *Pseudomonas* (PA-1) were isolated from rice and maize. Additionally, one Cr-tolerant strain of *Alcaligenes faecalis* (AF-1) was isolated from the rhizosphere of maize. A randomized group pot experiment with heavily Cr-contaminated (a total Cr concentration of 1020.18 mg kg^−1^) paddy clay soil was conducted and the effects of different bacteria on plant growth, absorption and accumulation of Cr in lettuce (*Lactuca sativa* var. Hort) were compared. The results show that: (i) the addition of SR-2, PA-1 and LB-5 could promote the accumulation of plant fresh weight by 10.3%, 13.5% and 14.2%, respectively; (ii) most of the bacteria could significantly increase the activities of rhizosphere soil catalase and sucrase, among which LB-1 promotes catalase activity by 224.60% and PA-1 increases sucrase activity by 247%; (iii) AF-1, SR-1, LB-1, SR-2, LB-2, LB-3, LB-4 and LB-5 strains could significantly decrease shoot the Cr concentration by 19.2–83.6%. The results reveal that Cr-tolerant bacteria have good potential to reduce shoot Cr concentration at the heavily contaminated soil and endophytic bacteria have the same or even better effects than rhizosphere bacteria; this suggests that bacteria in plants are more ecological friendly than bacteria in soil, thus aiming to safely produce crops in Cr-polluted farmland and alleviate Cr contamination from the food chain.

## 1. Introduction

Chromium (Cr) is a transition metal which widely exists in the earth’s crust [1], with natural average concentrations of 122 mg/kg [2], entering the ecosystem through weathering and rock leaching of Cr in chromite and other natural reservoirs [3]. Cr is widely used in leather, electroplating, chemical industry, mining, steel and other industries [4]. With the increase in usage, more than 30,000 tons of Cr emissions have been released into the environment globally over the past 50 years, making it gradually become a common environmental pollutant [5]. As a highly reactive element, Cr exists in nature in a variety of valence states, from −2 to + 6; among them, the most common valence states are trivalent chromium (Cr (III)) and hexavalent chromium (Cr (VI)) [6]. In the human body, Cr (III) is an indispensable element involved in normal glucose and lipid metabolism and is one of the essential trace elements [7]. The lack of Cr (III) can cause various diseases including atherosclerosis and diabetes. Meanwhile, Cr (III) in nature has the characteristics of low bioavailability and small migration. Different from Cr (III), Cr (VI) not only has high environmental solubility and migration, but also is a carcinogen in humans. It can cause respiratory diseases such as nasal mucosa ulceration, nasal septum perforation, laryngitis and gastrointestinal diseases [8,9], and is classified as a Class A human carcinogen by the United States Environmental Protection Agency [10]. When the high concentration of Cr (VI) in soil enters the food chain through the absorption channel of crops, it will exert its biological magnification property and cause damage [11]; meanwhile, it will cause pollution and damage to the environment and ecology and can even enter the human body and cause a series of diseases. In addition, Cr pollution has harmful effects on plant growth and crop yield. Cropland soil contaminated with Cr will interfere with the overall growth of crops and impair plant life activities, resulting in a reduction in crop yield and quality [12]. Normally, environmental Cr pollution refers to Cr (VI) contamination. Therefore, reducing Cr (VI) to Cr (III) or fixing it in soil through passivation have become the mainstream technologies of remediation methods for soil Cr pollution.

The continual increase in the human population coupled with the scarcity of new arable lands creates the need to explore polluted lands for food production and other useful endpoints [13]. At present, physical remediation methods (landfill, soil/solution washing, ultrafiltration, membrane filtration and excavation) and chemical remediation methods (advanced oxidation, precipitation, solvent extraction, ion exchange, chemisorption and reduction) have been developed to treat Cr pollution [14]. However, in Cr-polluted cropland, both chemical and physical remediation are usually economically unfeasible, and bring drawbacks on the long-term development and utilization of farmland [15]. Compared with chemical and physical remediation methods with high consumables, biological remediation has the characteristics of no damage to soil structure, little intervention to soil environment, no secondary pollution, relative safety, low cost (much lower than the other two methods) and convenient application; this has been gradually accepted by researchers and environmental protection workers in various countries [16].

The biological remediation methods of Cr-contaminated soil can be divided into three types: phytoremediation, microbial remediation and genetic engineering remediation [17]. Among them, microbial remediation methods have the advantages of being cost-effective, environmentally friendly and can improve soil fertility. Although Cr pollution reduces the number of microbial communities in the environment and inhibits the growth of Cr-sensitive microorganisms, it also causes Cr-tolerant microorganisms to proliferate and changes the species abundance [18]. A variety of Cr-tolerant bacteria including *Bacillus*, *Enterobacter*, *Pseudomonas*, *Streptomyces*, *Microbacilli*, *sulphurizing bacteria*, *Serratia* and *alkalogenic bacteria* have been found [19,20]; among them, *Bacillus cereus*, *Pseudomonas puticosa*, *Serratia marcescens* and other bacteria can remove 50–99% of Cr (VI) in the environment, according to the study of Elahi et al. [21]. These studies have demonstrated the feasibility of finding bioremediation materials from Cr-contaminated environments. However, the related microorganisms used for the bioremediation of Cr mainly come from Cr-contaminated soil and water [19,20]. For different types of Cr-contaminated soil, especially farmland soil with cash crops, it is still necessary to find more microbial resources [22]. Bacteria that can help plants relieve Cr stress in soil are mainly divided into rhizosphere bacteria and plant endophytic bacteria. Plant rhizosphere regions are affected by plant roots and are contained with many soil microorganisms, which participate in a series of complex biological and ecological processes and regulate plant physiology and morphology to a large extent. These microorganisms drive the cycle of nutrient elements in rhizosphere soil and play a key role in plant growth and development [23]. Currently, a fair amount of rhizosphere bacteria, such as Bifidobacterium thuringiensis, *Burkholderia* and *Pseudomonas*, have been found to be effective microbial inoculants for the phytoremediation of heavy metal pollution, significantly improving the heavy metal absorption capacity of plants [24,25].

Compared with rhizosphere bacteria, plant endophytic bacteria-mediated resistance to heavy metal stress has been recognized as one of the most successful microbial remediation technologies due to its ecological friendliness, cost-effectiveness and technical feasibility [26]. Endophytic bacteria are defined as microorganisms that perform all or part of their life activities in the tissues, organs and intercellular spaces of healthy plants without causing plant diseases and are important for plant growth and heavy metal absorption [27]. Additionally, endophytic bacteria have multiple biological functions, which makes them thought to be mutually beneficial and symbiotic with host plants; since they can reproduce within plant tissues, they may interact directly and closely with their hosts, facing less nutritional competition, in return for their host protecting them from adverse changes in the rhizosphere environment [28]. Studies have shown that endophytic bacteria can not only improve nutrient availability in plants, but also provide essential vitamins and growth conditions for plants [29]. In addition, endophytic bacterial communities have high variability, making them more susceptible to external endophytic inoculation. Thus, endophytes may show greater potential for phytoremediation than rhizosphere bacteria [30].

Studies on endophytic microorganisms removing Cr from the environment and helping host bioreactors include consortia of bacteria in *Lupinus luteus*, *Pseudomonas* sp. and *Microbaccterium* sp. in *Rumex acetosa*, *Mucorsp.* MHR-7 in *Brassica campestris* L. and *Serratia marcescens* PRE01 in *Pteris vittata* [31,32,33,34]. At present, in the process of microbial-assisted phytoremediation, a large number of rhizosphere microorganisms have emerged in an endless stream, but there are still few documented studies on endophytic microorganisms [35].

The research of Wang et al. [36] on inoculating different niche consortia concluded that the original ecological niches were not a major factor in the growth-promoting attributes, Cd phytoextraction efficiency and changes in bacterial community structure. Therefore, we proposed that endophytic bacteria could successfully colonize in the plant rhizosphere and have equally or better Cr remediation ability than rhizosphere bacteria; a pot experiment was conducted to prove the hypothesis. Lettuce is one of the most widely consumed vegetables with the highest economic value. According to the survey in the United States in 2018, the economic value created by lettuce exceeds 2.7 billion dollars and was chosen as the experimental subject [10]. Here, one strain of Cr-tolerant rhizosphere bacteria and eight different strains of Cr-tolerant endophytic bacteria were isolated from rhizosphere soil and plants of crops in Cr-contaminated farmland, and they were further inoculated on lettuce rhizosphere in a pot experiment with heavily Cr-polluted paddy soil. The objectives of this study were to: (1) study the effects of the strains on the variation in soil Cr and Cr (VI) concentration; (2) screen the strains that can reduce Cr accumulation in the edible part of lettuce; (3) compare the effects of endophytic bacteria with rhizosphere bacteria on soil Cr (VI) transformation and crop Cr uptake.

## 2. Materials and Methods

### 2.1. The Collection of Cr-Contaminated Soil and Plant Breeding

The soil was collected from Cr-contaminated paddy fields (121°21′5.75′′, 29°57′58.42′′) in Hemudu Town, Ningbo city, Zhejiang, China, at a depth of 0–20 cm, and the granulometric composition of the soil was clay. The average annual temperature in Hemudu Town was 16.2 °C, and the average annual precipitation was 2301.1 mm. Due to the illegal processing of stainless-steel hoses and dumping of industrial wastewater by the factory, the soil had been seriously polluted by Cr [37]. The soil samples were air dried and pulverized to 2 mm to carry out the pot experiment. The basic properties of the test soil were: pH 4.87, electrical conductivity (EC) 0.95 ds cm^−1^, organic matter (OM) 47.96 g kg^−1^, cation exchange capacity (CEC) 12.06 cmol kg^−1^, available nitrogen (AN) 0.11 g kg^−1^, available phosphorus (AP) 0.18 g kg^−1^, available potassium (AK) 0.19 g kg^−1^, total Cr 1020.18 mg kg^−1^, Cr (VI) 65.44 mg kg^−1^. Cr far exceeded the National Soil Environment Quality Standard of China (GB 15618–2018) for agricultural land (150 mg kg^−1^).

The lettuce (*Lactuca sativa* var. ramosa Hort) seed was purchased from Hangzhou seed company. In July 2022, seeds were sterilized with 2% H_2_O_2_, then rinsed with deionized water and planted in seeding trays. Then, the seedlings with consistent growth were selected for the pot experiment.

### 2.2. Isolation and Identification of Bacterial Strains

The isolation of rhizosphere bacteria: The Cr-tolerant bacteria were isolated from the rhizosphere of maize in heavily polluted farmland with a total Cr concentration of 404.90 mg kg ^−1^ in Xinchang Town, Shaoxing City (120°56′35.16′′, 29°15′19.33′′). A total of 1.0 g of soil sample was accurately weighed, put it into a triangle bottle containing 99.0 mL of sterile water and small glass beads and oscillated for about 20 min to disperse the microbial cells—this became the 10^−2^ soil sample diluent. A total of 1.0 mL of the diluent was absorbed with a sterile straw and transferred to a centrifuge tube containing 9.0 mL of sterile water; this was blown and sucked several times to ensure the bacterial solution was mixed evenly—this became the 10^−3^ diluent. This step was repeated to prepare a series of diluted bacterial solution up to 10^−6^. Then, the bacteria were smeared on beef extract peptone solid medium (beef extract 3 g L^−1^, peptone 10 g L^−1^, NaCl 5 g L^−1^) supplemented with 100–800 mg kg^−1^ Cr (VI), and the single colony was isolated, purified by a continuous streak, and then inoculated in the liquid medium, mixed 1:1 with 30% glycerin and stored at −80 °C in the refrigerator.

The isolation of endophytic bacteria: The Cr-tolerant endophytic bacteria were isolated from leaves and roots of maize/rice in Xinchang town and Hemudu town (2.1). Healthy plants (root/leaf tissue) were rinsed and soaked in 70% alcohol for 40 s under aseptic conditions, then continued to soak in 2.5% sodium hypochlorite solution for 2 min, and finally rinsed with sterile water several times until the disinfectant was completely removed. The sterile water of the last rinse was applied to the solid medium, and no microorganisms grew out after the culture could demonstrate the complete disinfection of plant surfaces. After adding 1 mL of the 0.9% NaCl solution, the disinfected plant tissue was fully ground, and 1 mL of the grinding solution was inoculated into a 500 mL triangle bottle containing 100 mL of beef extract peptone liquid medium. The culture medium was incubated at 37 °C and 120 r/min for 2 days and diluted according to the gradient of 10^−2^ to 10^−6^. The subsequent separation and screening operations were similar to the above paragraph.

The isolated strains were delivered to Tsingke Biotechnology Co., Ltd. for identification and sequencing. Sequences obtained using the BLAST search analysis from the National Center for Biotechnology Information (NCBI) database showed up to 99% or 100% similarity with different bacterial species. CLUSTALW was used to compare the result sequences in the NCBI database with relevant sequences.

### 2.3. Experimental Design

Ten days after germination, seedlings of a similar size were transplanted into each pot (two plants pot^−1^), and the N-P_2_O_5_-K_2_O compound fertilizer (15-15-15: 750 kg ha^−1^) was applied to ensure the normal supply of vegetable nutrients before transplantation. A pot experiment was carried out in the artificial climate chamber of Zijingang Campus, Zhejiang University. Each plastic pot (16 cm wide at top, 14 cm wide at bottom, and 12 cm in height) had a drainage hole at the bottom and was filled with 1.0 kg of soil. Nine selected bacteria were placed in the beef extract peptone liquid medium at 30 °C and 120 r min^−1^ for shock culture for 24 h, and the bacteria liquid OD value was adjusted to 1.0 (600 nm) by a spectrophotometer, in which the bacterial concentration was 2 × 10^9^ cfu mL^−1^ [38]. The pot experiment included the following ten different treatments: (1) Control (CK): adding 10 mL of the beef extract peptone liquid medium to the rhizosphere every 5 d; (2) (AF-1): Adding 10 mL of the rhizosphere bacteria AF-1 medium solution with an OD_600_ value of 1.0 to the rhizosphere every 5 d. (3)~(10) Experimental groups: adding 10 mL of the endophytic bacteria SR-1, SR-2, PA-1, LB-1, LB-2, LB-3, LB-4, LB-5 medium solution, respectively, with an OD_600_ value of 1.0 to the rhizosphere every 5 d. A total of 10 different treatments were used, and each treatment was repeated three times. There were 30 pots with 2 lettuce plants in each pot. The experiment pot was irrigated with deionized water and cultured for 3 w in a non-light environment. At the same time, it was randomly arranged in the artificial climate chamber using a completely randomized design. During the growth period, the addition of deionized water could keep soil moisture at about 70% of field capacity. Lettuce was harvested after growing in the pots for 45 d.

### 2.4. Plant Sample Analysis

The plant was gently pulled from the soil with its roots. The surface of the plant was repeatedly rinsed with deionized water, while the soil particles bound to the roots were cleaned and the surface moisture was absorbed with absorbent paper. Then, the plant was divided into two parts: the above-ground part (shoot) and the below-ground part (root). The fresh weight of the above-ground part was weighed on a balance. Then, the plant (including the shoots and roots) was dried in an oven at 65 °C for 4 days and was ground with a mortar until it was passed through a 0.15 mm sieve for subsequent chemical analysis. The concentration of Cr in the shoots and roots was determined by the acid digestion method: a total of 0.1 g of the plant samples was accurately weighed and put into a digest tube, adding 5 mL of concentrated nitric acid and 1 mL of hydrogen peroxide. After overnight, the samples were placed in a graphite furnace at 150 °C for digestion. After 4 h, 1 mL of hydrogen peroxide was added into each tube; this continued to digest until clear, and the transparent solution remained in the tube. The digest tube was then removed and cooled to room temperature. Then, the solution was weighed in the tube to 50 g with ultra-pure water. After filtration, 10 mL of the digestive solution was collected and the concentration of metal elements was determined by ICP-MS 7500a (Agilent, NY, USA).

Moreover, the chlorophyll concentration was determined by the acetone–ethanol extraction method: a total of 0.2 g of chopped fresh leaves was weighed and 10 mL of the extractant was added (80% acetone: 95% ethanol = 1:1; V/V); OD_645_ and OD_663_ were determined by a spectrophotometer after 24 h of light shelter treatment. The extraction agent was zeroed and the concentration of chlorophyll was calculated by the Arnon method’s modified formula [39]:

Ca = (12.71A663 − 2.59A645) × (V/M)(1)

Cb = (22.88A645 − 4.67A663) × (V/M)(2)

Ct = (8.04A663 + 20.29A645) × (V/M)(3)

In the above equation, Ca, Cb and Ct, respectively, represent the concentration of chlorophyll a, chlorophyll b and total chlorophyll; V is the volume of extracted liquid; M is the weight of the lettuce leaves taken.

### 2.5. Rhizosphere Soil Sample Analysis

The rhizosphere soil samples were taken out of the pot and air dried and screened into different particle sizes for subsequent determination.

Soil physical and chemical properties: soil pH was determined by the pH meter (PB-10, Sartorius, Germany) under the soil–water ratio condition of 1:5. Soil EC was determined by the Electrochemical Analyzer EC meter 4510 (Jenway, UK) after centrifugation (soil–water ratio of 1:5180 r min^−1^, 5 min). Soil CEC was determined by the CO(NH3)_6_Cl_3_ solution (1.66 × 10^−2^ M) with a soil–liquid ratio of 3.5:50 (HJ 889-017). The soil total OM was measured after being oxidized by K_2_Cr_2_O_7_-H_2_SO_4_ under external heating conditions. AN was evaluated by alkaline hydrolysable [40]. AP and AK were extracted with 0.5 M NaHCO_3_ and 1.0 M NH_4_Ac solutions, respectively, with soil–liquid ratios of 1:20 and 1:10 [41].

The soil Cr concentration: The concentration of total Cr in soil was determined by the standard acid digestion method adopted by Hamid et al. [42]. In brief, 0.1 g of the soil sample was weighed into a soil digest tube and 7 mL of the acidic mixture (HNO_3_, HClO_4_, HF; ratio of 5:1:1) was digested by a microwave digestion instrument for 2 h at 160 °C. The digestion solution was rinsed with ultra-pure water and filtered by a filter membrane, and the Cr concentration was determined by ICP-MS. The concentration of Cr (VI) in soil samples was determined by the alkali digestion method. A total of 0.25 g of soil samples was weighed into a round-bottled flask, and 5.0 mL of the alkali digestion solution (NaOH, Na_2_CO_3_; ratio of 2:3), 40 mg of MgCl_2_ and 0.05 mL of the buffer solution (K_2_HPO_3_, KH_2_PO_3_; ratio of 8.71:6.80) were placed on the constant temperature magnetic stirring heating device. After stirring for 5 min at a normal temperature, the samples were heated to 90~95 °C and then digested for 1 h. After the samples were digested, the samples were cooled to room temperature and filtered. The filtrate was placed in a 100 mL beaker, the pH was adjusted to 9.0 with concentrated nitric acid and the solution was transferred to a 100 mL volumetric bottle, and then was diluted to the scale with deionized water. After shaking, the concentration of Cr (VI) was determined by ICP-MS.

Soil enzyme activities: The study investigated the activities of catalase and sucrase in untreated and treated soils. Catalase activity was determined by the ultraviolet absorption method, and the activity was recorded as μmol (H_2_O_2_) d^−1^ g^−1^ [43]: a total of 2.0 g of the soil sample was poured into 40 mL of distilled water added with 5 mL of the H_2_0_2_ solution. After oscillating the treatment (25 °C, 200 r min^−1^, 20 min), 1 mL of aluminum–potassium alum was quickly added into the mixed solution and then filtered with 5 mL of 1.5 moL l^−1^ H_2_SO_4_. The absorbance of the filtrate at 240 nm was measured while making a comparison between soil-free and inorganic matter. Sucrase activity was measured using 3,5-Dinitrosalicylic acid colorimetry, while the obtained soil activity was presented as mg (glucose) g^−1^ d^−1^: a total of 5.0 g of soil was placed in a 50 mL triangle bottle filled with 15 mL of the 8% sucrose solution, 5 mL of the pH 5.5 phosphate buffer and 5 drops of toluene. Then, the mixed solution was oscillated and incubated in an incubator at 37 °C for 24 h. This was filtrated right after the incubation and 1 mL of the filtrate was absorbed and injected into a 50 mL volumetric bottle. Then, 3 mL of the DNS reagent was added, heated in a boiling water bath for 5 min, then the volumetric bottle was transferred to running water and cooled down for 3 min. The solution was orange-yellow due to the formation of 3-amino-5-nitrosalicylic acid. Finally, it was diluted to 50 mL with distilled water and colorimetric at 510 nm on a spectrophotometer. In order to eliminate the errors caused by sugar and glucose in the soil, no matrix control should be made for each soil sample, and no soil control should be made for the whole test; if the absorption value of the sample exceeds the maximum value of the scale curve, the fractional ratio should be increased or the soil sample cultured should be reduced. Both enzyme activity assay kits were purchased from Suzhou Comin Biotechnology Co., Ltd.

### 2.6. Quality Control

Certified standard reference materials for plant (GBW (E) 100495) and soil (GBW07917) and three reagent blanks were employed to ensure the accuracy and precision of the experimental results. The recovery rates of Cr in plants and soils were 95.7 ± 5.2% and 98.2 ± 8.9%, indicating that our data were reliable because the values conformed to the error range <10%.

### 2.7. Translocation Factor (TF) and Bioaccumulation Factor (BAF)

The translocation factor (TF) from root to shoot was reckoned using Equation (4), as recorded by [42]:

(4) $$\mathrm{TF}=\frac{C_{Shoot}}{C_{Root}}$$

The bioaccumulation factor (BAF) from soil to plant was calculated by Equation (5), as described by Khan [44]:

(5) $$\mathrm{BAF}=\frac{C_{Shoot}}{C_{Soil}}$$

where *C_Shoot_* refers to the concentration of Cr in the shoot of lettuce, *C_root_* refers to the concentration of Cr in root of lettuce and *C_Soil_* refers to the concentration of total Cr in soil on a dry weight basis.

### 2.8. Statistical Analysis

Differences of the treatments were compared using one-way ANOVA at a significance level of *p* < 0.05 by SPSS 20.0 software. All results were expressed as an average value of three replicates ± standard error. Origin 2023b was used to plot graphs. The correlation analysis between lettuce growth, physicochemical features, enzyme activities and Cr speciation was completed via Origin 2023b.

## 3. Results

### 3.1. Isolation and Identification of Cr-Tolerant Bacteria

After the isolation experiment, four rhizosphere bacterial colonies in the soil, three endophytic bacteria colonies in roots and two endophytic bacteria colonies in leaves were isolated from maize in Xinchang County, and nine endophytic bacteria colonies in leaves and eight endophytic bacteria colonies in roots were isolated from maize and rice in Yuyao City. Bacteria capable of removing Cr (VI) from the environment were further screened. After sequencing analysis, nine strains of Cr-tolerant bacteria were finally screened out for the follow-up experiment, including one strain of rhizosphere bacteria: AF-1 (*Alcaligenes faecalis*); and eight strains of endophytic bacteria: SR-1/SR-2 (*Serratia*), LB-1/LB-2, LB-3/LB-4/LB-5 (*Lysinebacillus*), PA-1 (*Pseudomonas aeruginosa*) (Table 1).

### 3.2. Influence of Bacteria on Rhizosphere Soil Physiochemical Properties

In the CK treatment, soil pH was 4.78, EC: 1.08 ds cm^−1^, CEC: 8.98 cmol kg^−1^, OM: 29.06 g kg^−1^, AN: 114.08 mg kg^−1^, AP: 164.98 mg kg^−1^ and AK: 151.23 mg kg^−1^ (Table 2). Different types of Cr-tolerant bacteria significantly increased the pH value of rhizosphere soil. The maximum increase in soil pH was observed after SR-2 was sprayed, which was significantly increased by 7.11%, followed by treatments of LB-3, LB-1 and LB-2 (Table 2). For soil EC, SR-2 obviously reduced by 4.63%, whereas there were no significant differences in other treatments (Table 2).

Bacteria spraying could significantly promote soil CEC, except PA-1 strain (Table 2). The addition of *lysine bacillus* LB-5, LB-4 and LB-3 had the most significant promoting effect on soil CEC, increasing by 18.4%, 11.6% and 8.80%. The addition of LB-1 significantly increased the soil organic matter concentration to 31.84 g kg^−1^ by 9.57%, but other bacteria had no obvious effect on it (Table 2).

The addition of bacteria also significantly increased the soil AN concentration (Table 2). Soil AN concentrations under LB-4 and LB-5 treatments were the highest, which increased by 56.5% and 53.2%; followed by treatments of LB-1, SR-2 and LB-3 with an increase of 52.3%, 48.94% and 45.6%; and then treatments of AF-1, SR-1 and LB-2 with an increase of 40.7%, 37.6% and 27.9%. The improvement effect of PA-1 treatment on soil AN concentration was the weakest with an increase of 13.3%. Treatment PA-1 significantly increased the soil AP concentration with an increase of 11.0%, while other treatments had no obvious change on it (Table 2). The soil AK concentration increased most significantly in LB-4, LB-2, SR-1 and SR-2, ranging from 12.9 to 21.8%, followed by LB-5, LB-3, AF-1, LB-1 and PA-1, with a range of 4.53–10.1% (Table 2).

### 3.3. Soil Enzyme Activities

**Figure 1:**
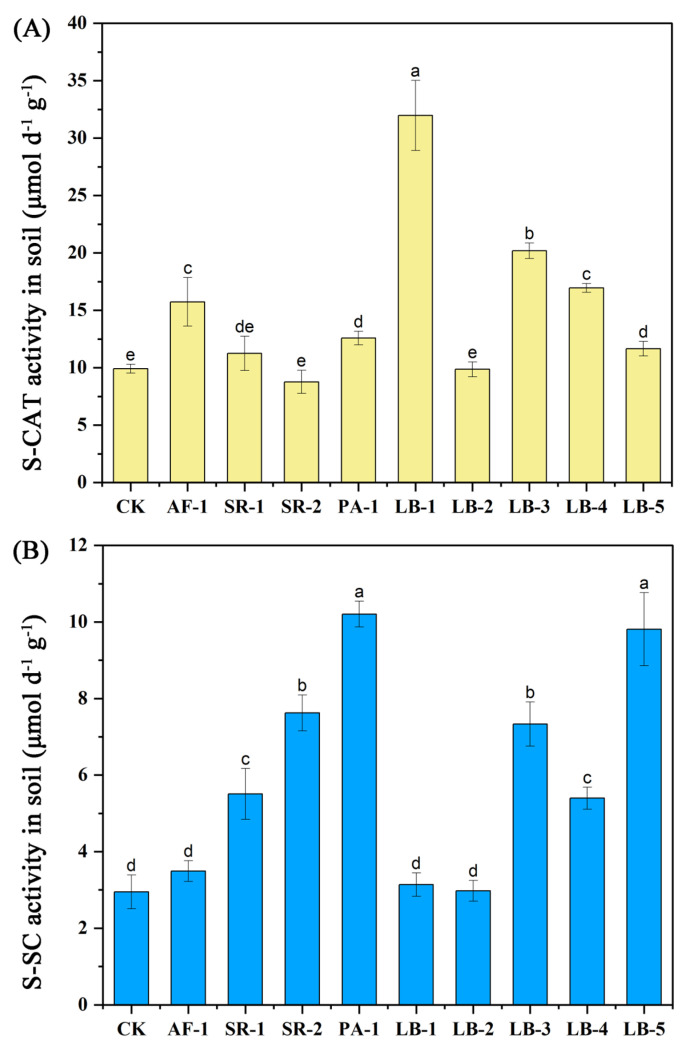
S-CAT and S-SC activity in the rhizosphere soil of different treatments. (**A**): S-CAT activity in soil. (**B**) S-SC activity in soil. The same letter means no significant difference.

The soil enzyme activities (catalase and sucrase) were significantly affected by the addition of different bacteria (Figure 1). The catalase activity in CK was 9.91 μmol d^−1^ g^−1^. However, no significant changes were observed in SR-2 and LB-2. The addition of other Cr-tolerant bacteria could increase soil catalase activity to varying degrees; among them, LB-1 treatment was the highest, and increased by 222.60%. The increasing rate of the catalase activity of other bacteria, from high to low, were LB-3 (103.7%), LB-4 (71.0%), AF-1 (58.7%), PA-1 (26.8%), LB-5 (17.6) and SR-1 (13.5%).

After the addition of AF-1, LB-1 and LB-2, the sucrase activity of lettuce rhizosphere soil did not change significantly compared with CK (2.94 μmol d^−1^ g^−1^), while SR-1, SR-2, LB-6 and LB-5 all enhanced the sucrase activity of soil to varying degrees. In terms of increasing range, PA-1 (247.3%) and LB-5 (233.7%) had the most obvious strengthening effect, followed by SR-2 (161.86%), LB-3 (159.18%), SR-1 (87.4%) and LB-4 (84.2%).

### 3.4. Soil Total Cr and Cr (VI) Concentration

**Figure 2:**
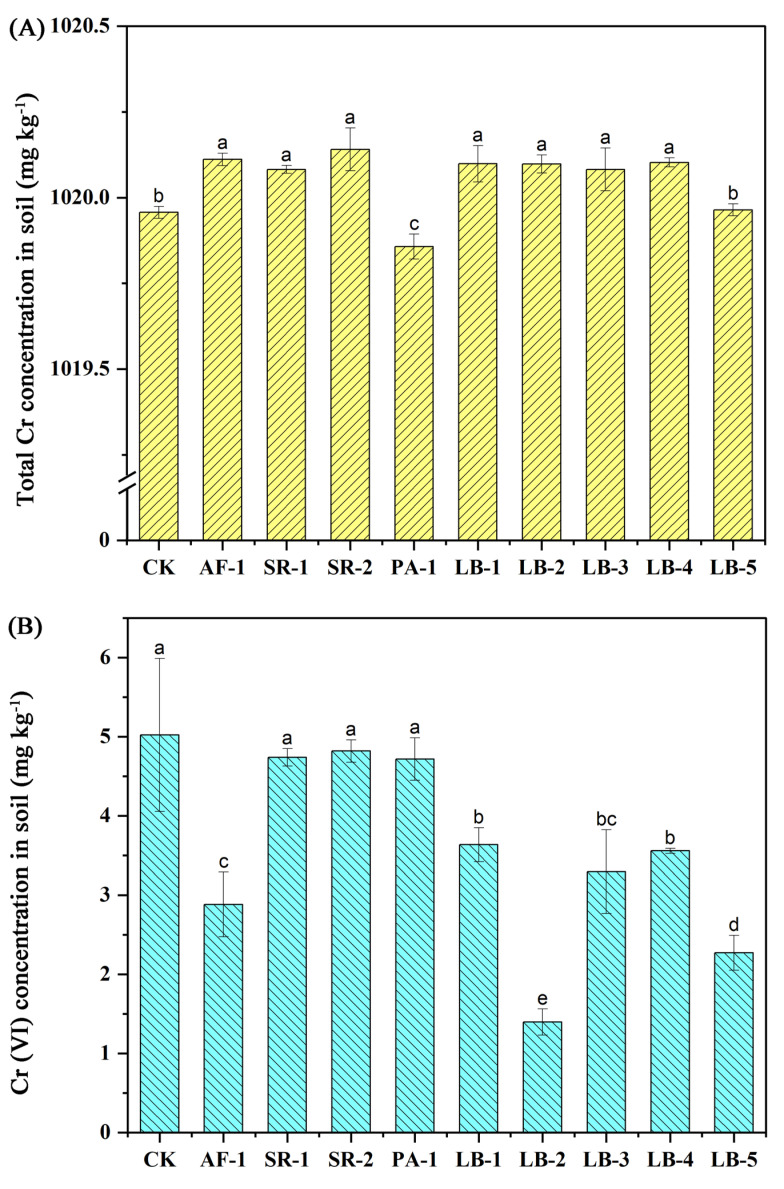
Total Cr and Cr (VI) concentration in the rhizosphere soil of different treatments. (**A**): Total Cr concentration in soil; (**B**): Cr (VI) concentration in soil. The same letter means no significant difference.

After the plants were harvested, except the addition of PA-1 and LB-5, all the treatments could reduce the extraction of Cr from the plant, resulting in a significantly higher total Cr concentration in the soil than in CK (Figure 2A). Among them, the total Cr concentration in soil under the SR-2 treatment was the highest, with an increase of 0.18 mg kg^−1^ compared with CK (1019.96 mg kg^−1^), followed by AF-1, LB-4, LB-1, LB-2, LB-3, SR-1, which ranged between 0.12 and 0.15 mg kg^−1^.

There was no significant change in soil Cr (VI) concentration after being treated with SR-1, SR-2 and PA-1 (Figure 2B), while the Cr (VI) concentration in treatment LB-2 decreased by 70.5% compared with CK, followed by LB-5, which decreased by 54.8%, then AF-1 and SR-2 with decrease values of 42.6% and 27.5%.

### 3.5. Plant Growth and Photosynthetic Pigments

**Figure 3:**
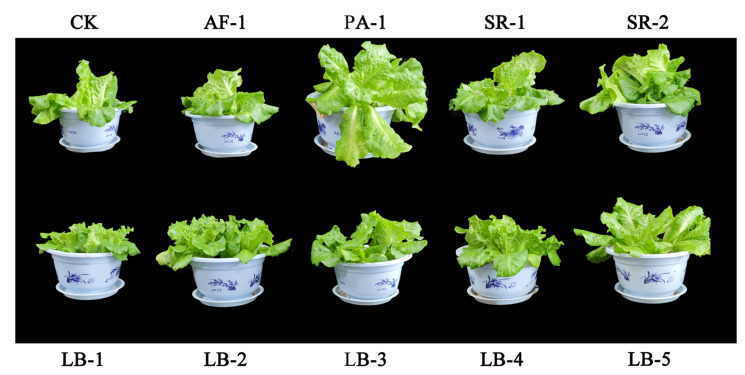
The growth of lettuce in different treatments.

The average fresh weight per plant of CK was 24.27 kg. Except for treatment LB-1, which had a significantly lower value of fresh weight than it, all other treatments showed no obvious change (Table 2; Figure 3).

In CK, the concentrations of plant chlorophyll a, b and total chlorophyll were 428.73 mg kg^−1^, 139.27 mg kg^−1^ and 568.00 mg kg^−1^ (Table 3), respectively. We compared with them the concentrations of chlorophyll which increased the most under treatment SR-2, reaching 86.3% (chlorophyll a) and 82.0% (chlorophyll b), followed by AF-1, which increased by 34.8% (chlorophyll a) and 33.8% (chlorophyll b). There were no significant changes after the addition of LB-1, PA-1 and LB-4. Surprisingly, chlorophyll a and b concentrations in shoots treated with SR-1, LB-2, LB-3 and LB-5 all decreased obviously.

### 3.6. Cr Uptake and Accumulation in Plant

**Figure 4:**
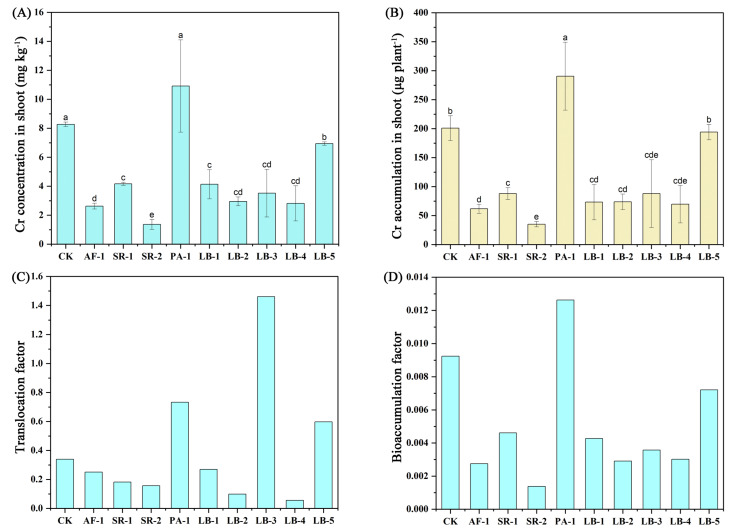
The Cr uptake, translocation and bioaccumulation of plants in different treatments. The data are average of three replicates ± SE. (**A**): Cr concentration in shoot; (**B**): Cr accumulation in shoot; (**C**): Translocation factor; (**D**): Bioaccumulation factor. The same letter means no significant difference.

Except for PA-1, all treatments with Cr-tolerant bacteria could significantly reduce the Cr concentration in the plant shoot (Figure 4). Among them, treatment SR-2 had the best effect, with a decrease of 83.6%, followed by AF-1, LB-4, LB-2, LB-3, LB-1, SR-1, LB-5, with a decrease range of 19.2~68.2%. As for Cr accumulation, all treatments except for PA-1 and LB-5 significantly reduced the Cr accumulation in lettuce per plant. SR-2 had the best effect, reducing it by 82.5%. Furthermore, AF-1, LB-4, LB-1, LB-2, LB-3 and SR-1 had, respectively, decreased Cr accumulation by 69.3%, 65.3%, 63.5%, 63.3%, 56.2% and 56.2%.

TF and BF values increased significantly under PA-1 treatment, and TF values under LB-3 and LB-5 treatment also had a significant improvement. Both TF and BAF values of other Cr-tolerant bacteria were decreased, with reduction ranges of TF (26.0–85.3%) and BAF (53.8–70.6%), among which LB-4 had the lowest TF and AF-1 had the lowest BAF (Figure 4).

### 3.7. Correlation Analysis between Soil Environmental Variables and Plant Indexes

**Figure 5:**
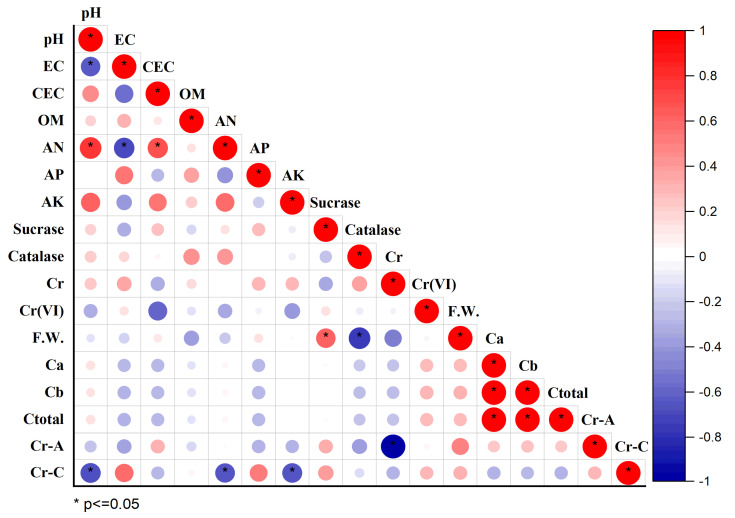
Correlation analysis of basic physicochemical properties, enzyme activities, Cr bioavailability in rhizosphere soil and Cr bioaccumulation in plant. Cr: soil total Cr content, Cr (VI): soil Cr (VI) content, F.W.: fresh weight, Ca: chlorophyll a, Cb: chlorophyll b, Ctotal: total chlorophyll, Cr-A: Cr accumulation in shoot, Cr-C: Cr concentration in shoot.

A correlation analysis of soil environmental variables and plant indexes is used in Figure 5 to uncover the important characteristics influencing soil Cr mobility, absorption and accumulation in lettuce. Soil pH was significantly positively correlated with soil AN and soil total Cr concentration (*p* < 0.05)—the Pearson correlation coefficient^®^ was 0.78 and 0.66; it was significantly negatively correlated with soil EC (r = −0.64) and Cr concentration in the shoot of lettuce (r = −0.69) (*p* < 0.05). Soil CEC concentration was positively correlated with soil AN concentration (r = 0.69), and negatively correlated with soil Cr (VI) concentration (r = −0.62). Soil fresh weight was positively correlated with soil sucrase activity (r = 0.63), and negatively correlated with soil catalase activity (r = −0.78). There was a significant negative correlation between the total Cr concentration and Cr (VI) concentration in soil treated by different Cr-tolerant bacteria, and the Pearson correlation coefficient was −0.72. The Cr concentration in the shoot of lettuce was negatively correlated with soil AN concentration, soil AK concentration and soil total Cr concentration—the correlation coefficients were −0.66, −0.69 and −0.70, respectively (*p* < 0.05). Meanwhile, the total Cr concentration in soil was the most important factor affecting the accumulation of Cr in edible lettuce (r = −0.99), which conformed to the total Cr balance in the plant and soil envieronment.

## 4. Discussion

### 4.1. Influence of Cr-Tolerant Bacteria on Soil Physicochemical Properties

Soil pH has an important influence on the presence of heavy metals in soil environments [45]. Heavy metals have high solubility, fluidity and bioavailability at low pH values; with an increase in pH, the form of heavy metals tends to become stable [46]. Shahid et al. [47] also confirmed that a decrease in the Cr (VI) concentration in soil can cause an increase in the soil pH value. In this study, the selected bacteria significantly increased the pH of the heavily Cr-contaminated paddy soil (Table 2), which was consistent with previous research results. Therefore, the passivation of Cr-tolerant bacteria causes an increase in soil pH; the increase in pH can also promote the stability of soil Cr, forming a benign continuous passivation of Cr in the soil system. In addition, the experimental results also showed that the addition of Cr-tolerant bacteria on soil nutrition was mainly reflected in the significant increase in soil CEC, AN, AK concentrations. These indexes can represent soil fertility, crop growth and pollutant transport capacity, and are important parameters for predicting crop yield [48,49].

### 4.2. Influence of Cr-Tolerant Bacteria on Soil Enzyme Activities

According to the study of Thorgersen et al. [50] and Hu et al. [12], excessive Cr in soil can affect the metabolic function of the microbial community by inhibiting metabolic pathways and functional genes (especially those involved in denitrification), so Cr pollution leads to a decrease in soil microbial diversity. The level of enzyme activity in soil can be used as an important index to detect the change in soil microbial function and is important evidence for the immobilization of heavy metals in soil [51,52,53]. Catalase can split hydrogen peroxide into molecular oxygen and water to prevent cells from damage by reactive oxygen species—the activity of catalase in soil is related to the metabolic activity of aerobic organisms and has been used as an indicator of soil fertility [54,55]. The effect of sucrase in soil is closely related to soil organic matter metabolism and nitrogen and phosphorus concentration while its activity is also an index to soil fertility levels [56]. Previous research [57,58,59,60] confirmed that *Bacillus, Pseudomonas, Serratia marcescens* and *Alcaligenes faecalis* all had great colonization in heavily Cr-polluted soil and could become the dominant bacterial community and increase the abundance of microorganisms in soil, thereby improving soil environmental conditions, thus providing strong support for this study. Soil catalase activity and sucrase activity increased significantly after being treated with the majority of Cr-tolerant bacteria (Figure 1); among them, endophytic bacteria PA-1, LB-3, LB-4 and LB-5 could simultaneously promote the activity of catalase and sucrase—this result not only supported the improvement in soil fertility (Table 2), but also proved that the addition of endophytic bacteria had a better effect than rhizosphere bacteria on increasing the physiological activities of microorganisms in Cr-contaminated soil.

### 4.3. Influence of Cr-Tolerant Bacteria on Lettuce Growth and Photosynthetic Pigments

The improvement in soil nutrition did not significantly increase the fresh weight of lettuce. According to previous experimental data [61], the average fresh weight per plant of this variety of lettuce ranges from 16.50 to 35.10 kg with or without the addition of various biochar and compost treatment, while the fresh weight of the lettuce in this experiment ranged from 16.90 to 27.92 g (Table 3). This phenomenon indicated that the Cr-tolerant bacteria used in this study did not directly promote the growth of lettuce, although it could improve the nutrient level of Cr-contaminated soil.

As for photosynthetic pigments, Christou et al. [62] studied the effects of Cr (VI) stress on the photosynthetic pigment concentration and oxidative stress markers of lettuce plants; they found that the chlorophyll concentration of lettuce plants increased with the increase in environmental Cr stress after being treated with Cr (VI) containing solution in the range of 0.05–10 mg/L. Combined with the experiment of Agathokleous et al. [63], it was concluded that plants exert their own antagonism by accumulating and increasing photosynthetic pigment concentration in leaves under mild, heavy metal stress. The potting soil in this study was heavily Cr-contaminated, and the chlorophyll concentrations were at high levels on all treatments; however, there was no significant increase or decrease relationship with CK, which was also proven by the insignificant correlation between the level of photosynthetic pigment and soil physicochemical properties/enzyme activities/each parts’ Cr concentration or plant fresh weight (Figure 5).

### 4.4. Influence of Cr-Tolerant Bacteria on Soil Total Cr Concentration and Soil Cr (VI) Concentration

Pushkar et al. [64] explored the remediation of Cr (VI) by different types of microorganisms in their review on the remediate mechanism of Cr-contaminated microorganisms, and believed that microorganisms could complete the remediation of Cr (VI) contamination through cell surface interaction, extracellular polysaccharide action, direct reduction and efflux mechanisms. In this study, most endophytic bacteria could significantly reduce the concentration of Cr (VI) in soil, promote the passivation and accumulation of Cr in soil, and thus reduce the migration of Cr in Cr-contaminated soil (Figure 2), which were consistent with expectations. Similar results can be obtained from the previous literature: Chai et al. [65] isolated strain *Pannonibacter phragmitetus* sp. from Cr-contaminated soil, which could effectively remove Cr (VI) in soils with a Cr concentration of 25–500 mg L^−1^. Similarly, strain *Cupriavidus* sp. was isolated from tropical agricultural soils by Minari et al. [66], and could effectively remove 60% of the Cr (VI) in the medium. In addition, the *Bacillus cereus*, *Bacillus licheniformis* and *Bacillus subtilis* of *Bacillus genus* all have good Cr (VI) removal ability in solutions, and the removal rates were 100%, 95% and 93.5% [16,35,67]. In this study, strains AF-1, LB-1, LB-2, LB-3, LB-4 and LB-5 had significantly decreased rhizosphere soil Cr (VI) concentrations from 27.5% to 70.5%, indicating the Cr (VI)-reducing ability of these bacteria. In addition, compared with the rhizosphere bacteria AF-1, endophytic bacteria SR-1, SR-2, LB-1, LB-2, LB-3 and LB-4 had similar effects on the passivation of total Cr in rhizosphere soil. In terms of removing Cr (VI) in rhizosphere soil, endophytic bacteria LB-3 had the same ability as rhizosphere bacteria AF-1, while endophytic bacteria LB-2 and LB-5 were significantly stronger, which proved that the different separation sources and colonization sites of Cr-tolerant bacteria did not affect their promotion of soil Cr remediation.

### 4.5. Influence of Cr-Tolerant Bacteria on Cr Concentration in Lettuce

The Cr concentration in plant shoots and the Cr accumulation were significantly decreased after different treatments of bacteria, except for PA-1 and LB-5, while effectively inhibiting the transport activities of Cr in soil–lettuce systems (Figure 4). Shameer and Prassad [68] considered that rhizosphere microbial and plant interactions play an important role in enhancing plant repair potential through a mechanism called “bio-assisted phytoremediation”. The ability of plants to absorb Cr depends on the characteristics of different plant species and soil Cr morphology [69,70]. Compared with Cr (III), Cr (VI) has a higher soil–plant transfer index [71], which is more likely to lead to an increase in Cr concentration in plants. Therefore, one of the reasons that caused the decrease in Cr concentration in plant shoot was the removal of Cr (VI) from the soil by bacteria (4.4), which can be proven by the correlation analysis in Figure 5, whereby the Cr concentration in plant shoot was positively correlated with the concentration of Cr (VI) in soil (r = 0.33). In addition, there was a significant negative correlation between Cr accumulation in the plant shoot and total Cr concentration in rhizosphere soil (r = −0.99). This result not only confirmed the balance of total Cr concentration in soil–plant systems, but also indicated that the bacteria could reduce the absorption of Cr by plants by passivating Cr to achieve the purpose of safe utilization in heavily Cr-polluted paddy soil. There are many studies on the safe production of plants assisted by microbials in Cr-contaminated soil. The authors of [72] found that after adding *micrococcus luteus* to Cr-contaminated soil, the Cr concentration in the shoot of maize decreased by 65.2%; the inoculation of *Pseudomonas* into the rhizosphere of *Medicago sativa* could also increase plant resistance to Cr stress and improve plant growth [73]; and the study of Upadhyay et al. [74] confirmed the plant growth promotion and Cr repair ability of *Bacillus subtilis* as well. At the same time, the reduction in Cr (VI) and fixation of Cr (III) in the rhizosphere environment were completed, which alleviated the stress of Cr in crops. Currently, *Bacillus subtilis* has become the mainstream choice for the remediation of plant root nodules in Cr-contaminated soil. The above research results were highly consistent with the results of this experiment and were mutually verified.

According to the Limit of Pollutants in Food under the National Standard for Food Safety (2017), the limit of Cr in vegetables is 0.5 mg kg^−1^. Using the 90% water content of lettuce as a baseline, the lettuce treated with AF-1, SR-1, SR-2, LB-1, LB-2, LB-3 and LB-4 could achieve the safe consumption standards, while others could not. Therefore, the passivation effect of Cr-tolerant bacteria could effectively assist lettuce to complete Cr safety production in such soil. As for bacteria from different isolation sources, endophytic bacteria showed similar remediation ability as rhizosphere bacteria AF-1; among them, SR-2 even had better remediation capability than AF-1. This result indicated that the endophytic bacteria could exert similar or even better remediation ability in plant rhizosphere than original rhizosphere bacteria. The research of Wang et al. [36] on inoculating different niche consortia concluded that the original ecological niches were not a major factor in the growth-promoting attributes, Cd phytoextraction efficiency and changes in bacterial community structure, thus suggesting that ecological niche was not the primary determinant for the effective bioaugmentation inoculant construction, which was consistent with the results of this study.

## 5. Conclusions

The results of this study indicated that rhizosphere and endophytic Cr-tolerant bacteria inoculated in the Cr-polluted rhizosphere soil could effectively improve lettuce rhizosphere soil fertility by increasing soil pH, AN, AK, and the activities of sucrase and catalase. It was also confirmed that these bacteria have the ability to reduce the accumulation of Cr from the rhizosphere to plant shoots. Among them, treatment SR-2 has the most obvious effect, reducing the concentration of Cr in the edible part of lettuce by 83.92%. In the meantime, Cr-tolerant bacteria could also reduce the concentration of Cr (VI) in rhizosphere soil; among which, LB-2 has the highest decrease rate of 70.5%. In addition, the isolated source of bacteria is not a decisive factor in determining their remediate function in rhizosphere soil, endophytic bacteria (SR-1, LB-1, SR-2, LB-2, LB-3 and LB-4) have the same or even better effect than rhizosphere bacteria (AF-1), while all of them could ensure the safe utilization of lettuce in heavily Cr-polluted farmlands.

## Figures and Tables

**Table 1:** Characteristics of nine different Cr-tolerant bacteria isolated from the rhizosphere soil and plant of crops in Cr-contaminated farmland.

Name of Bacteria	Species	Scientific Name	Source Area	Host Plant	Separation Site	MIC * (mg kg^−1^)	Cr (VI) * Removal Rate
AF-1	*Alcaligenes*	*Alcaligenes faecalis*	Xinchang	maize	Rhizosphere soil	800	99.8%
SR-1	*Serratia*	*Serratia* sp.	Xinchang	rice	leaf	400	99.6%
SR-2	*Serratia*	*Serratia nematodiphila*	Yuyao	rice	root	400	71.1%
PA-1	*Pseudomonas*	*Pseudomonas aeruginosa*	Yuyao	maize	leaf	400	99.5%
LB-1	*Lysinebacillus*	*Lysinibacillus* sp. *Strain SePC-36*	Xinchang	rice	root	800	77.0%
LB-2	*Lysinebacillus*	*Lysinibacillus mangiferihumi strain WK63*	Yuyao	rice	leaf	800	99.8%
LB-3	*Lysinebacillus*	*Lysinibacillus sphaericus strain HBUM07034*	Yuyao	rice	root	800	99.8%
LB-4	*Lysinebacillus*	*Lysinibacillus magniferhumi strain M-GX18*	Yuyao	rice	leaf	800	99.9%
LB-5	*Lysinebacillus*	*Lysinibacillus* sp. *Strain M-3*	Yuyao	rice	root	800	99.8%

* MIC: minimum Cr (VI) inhibitory concentration; Cr (VI) removal rate: removal rate of Cr (VI) by Cr-tolerant bacterium at a beef extract peptone solution medium supplemented with 300 mg L^−1^ Cr (VI) in 24 h.

**Table 2:** The influence of different treatments on soil pH, EC, CEC, OM, AN, AP and AK.

Item	pH	Electric Conductivity (ds cm^−1^)	Cation Exchange Capacity (cmol kg^−1^)	Organic Matter (g kg^−1^)	Available Nitrogen (mg kg^−1^)	Available Phosphorus (mg kg^−1^)	Available Potassium (mg kg^−1^)
CK	4.78 ± 0.05 d	1.08 ± 0.04 a	8.98 ± 0.08 f	29.06 ± 0.47 b	114.02 ± 2.49 d	164.98 ± 3.75 b	151.24 ± 1.60 c
AF-1	5.00 ± 0.03 bc	1.05 ± 0.07 ab	9.13 ± 0.06 e	29.05 ± 0.28 b	160.43 ± 1.56 b	165.74 ± 5.38 b	165.77 ± 4.17 b
SR-1	5.03 ± 0.05 b	1.05 ± 0.04 ab	9.51 ± 0.03 d	30.07 ± 0.44 b	156.84 ± 8.16 b	167.80 ± 6.50 b	175.38 ± 3.83 ab
SR-2	5.12 ± 0.03 a	1.03 ± 0.01 b	9.55 ± 0.08 d	29.43 ± 1.32 b	169.82 ± 18.50 ab	164.54 ± 6.92 b	170.74 ± 4.61 ab
PA-1	4.96 ± 0.02 c	1.08 ± 0.02 a	8.97 ± 0.20 ef	30.54 ± 0.68 b	129.21 ± 4.12 c	183.09 ± 2.90 a	158.09 ± 1.54 b
LB-1	5.05 ± 0.03 b	1.07 ± 0.05 ab	9.61 ± 0.11 cd	31.84 ± 0.28 a	173.65 ± 18.61 ab	170.10 ± 4.88 b	163.09 ± 3.22 b
LB-2	5.05 ± 0.05 ab	1.07 ± 0.05 ab	9.87 ± 0.32 b–d	30.74 ± 1.93 ab	145.88 ± 14.90 bc	175.34 ± 12.62 ab	179.10 ± 3.45 ab
LB-3	5.07 ± 0.03 ab	1.05 ± 0.04 ab	9.77 ± 0.08 c	28.60 ± 1.10 b	166.07 ± 4.55 ab	169.92 ± 6.46 b	165.04 ± 5.19 b
LB-4	5.01 ± 0.02 b	1.06 ± 0.08 ab	10.02 ± 0.16 b	30.29 ± 1.07 b	178.39 ± 8.24 a	162.74 ±2.23 b	183.29 ± 5.95 a
LB-5	5.01 ± 0.0.3 b	1.04 ± 0.05 ab	10.63 ± 0.10 a	29.73 ± 2.04 ab	174.71 ± 7.25 a	166.46 ±5.71 b	166.54 ± 4.15 b

Note: The data are the average of three replicates ± SE. The different letters in the same column mean a significant difference at *p* < 0.05.

**Table 3:** The fresh weight, chlorophyll a, b and total chlorophyll content of lettuce as affected by different Cr-tolerant bacteria.

Treatments	Fresh Weight (g/plant)	Chlorophyll a (mg kg^−1^)	Chlorophyll b (mg kg^−1^)	Total Chlorophyll (mg kg^−1^)
CK	24.27 ± 2.51 a	428.73 ± 46.92 c	139.27 ± 12.21 cd	568.00 ± 59.13 c
AF-1	23.55 ± 3.08 ab	577.98 ± 96.77 b	186.33 ± 14.57 b	764.31 ± 111.34 b
SR-1	21.10 ± 1.96 ab	261.94 ± 38.85 e	91.71 ± 13.84 de	353.66 ± 52.69 d
SR-2	26.78 ± 4.55 a	798.81 ± 76.50 a	253.44 ± 15.64 a	1052.25 ± 92.14 a
PA-1	27.55 ± 3.64 a	414.04 ± 52.32 cd	138.06 ± 18.96 cd	552.11 ± 71.28 c
LB-1	16.90 ± 4.23 b	389.06 ± 42.16 cd	123.05 ± 21.20 d	512.11 ± 63.36 c
LB-2	24.76 ± 2.06 a	345.69 ± 24.55 d	108.49 ± 13.99 d	454.18 ± 38.54 cd
LB-3	22.72 ± 4.96 ab	215.46 ± 61.47 e	70.35 ± 16.48 e	285.81 ± 77.95 d
LB-4	24.19 ± 6.70 ab	446.63 ± 48.96 c	146.42 ± 11.10 c	593.05 ± 60.06 bc
LB-5	27.92 ± 1.43 a	316.34 ± 52.00 d	109.07 ± 14.66 d	425.41 ± 66.66 cd

Note: The data are the average of three replicates ± SE. The different letters in the same column mean a significant difference at *p* < 0.05.

## Data Availability

The data presented in this study are available on request from the corresponding author.
